# Retromer and the cation‐independent mannose 6‐phosphate receptor—Time for a trial separation?

**DOI:** 10.1111/tra.12542

**Published:** 2017-12-21

**Authors:** Matthew N. J. Seaman

**Affiliations:** ^1^ Cambridge Institute for Medical Research, Addenbrookes Hospital University of Cambridge Cambridge UK

**Keywords:** cargo‐selection, CIMPR, endosome‐to‐Golgi retrieval, Retromer, SNX‐BAR, sorting motif

## Abstract

The retromer cargo‐selective complex (CSC) comprising Vps35, Vps29 and Vps26 mediates the endosome‐to‐Golgi retrieval of the cation‐independent mannose 6‐phosphate receptor (CIMPR). Or does it? Recently published data have questioned the validity of this long‐established theory. Here, the evidence for and against a role for the retromer CSC in CIMPR endosome‐to‐Golgi retrieval is examined in the light of the new data that the SNX‐BAR dimer is actually responsible for CIMPR retrieval.

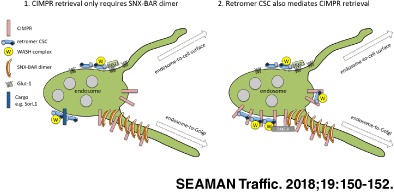

## 1 INTRODUCTION

Since it was first characterized nearly 20 years ago there has been a panoply of published reports detailing the functioning of the retromer complex in trafficking a varied array of membrane proteins from endosomes to either the Golgi complex or the cell surface.^1^, ^2^ One of the most widely accepted tenets of retromer function is that, in mammalian cells, the retromer complex mediates the endosome‐to‐Golgi retrieval of the cation‐independent mannose 6‐phosphate receptor (CIMPR) through the association of the cytoplasmic tail of the CIMPR with the cargo‐selective complex (CSC) of retromer comprising the Vps35, Vps29 and Vps26 proteins. Now though, this has been thrown into doubt after studies from Cullen and Steinberg report that the retromer CSC does not associate with the tail of the CIMPR and is not required for the endosome‐to‐Golgi retrieval of the CIMPR. Rather, the SNX‐BAR dimer, comprising SNX1 or SNX2 with either SNX5 or SNX6 associate with the CIMPR to mediate its retrieval.^3^, ^4^

## 2 SOME HISTORY

How and why was the retromer CSC believed to mediate the endosome‐to‐Golgi retrieval of the CIMPR for so long if that theory is wrong? The first reports that the retromer CSC was required to sort the CIMPR for retrieval to the Golgi came from studies independently conducted in the labs of Bonifacino and myself.^5^, ^6^ But those studies followed on from the initial work in yeast which favoured a direct association of the retromer CSC with cargo proteins, such as Vps10p.^7^, ^8^ As Vps10p in yeast performs the same task as the CIMPR in mammalian cells, it seemed natural that the retromer CSC would associate with the tail of the CIMPR and mediate its endosome‐to‐Golgi retrieval. Indeed the study from the Bonifacino lab reported a direct interaction between Vps35 and the CIMPR tail. Both studies reported that the CIMPR requires the function of the retromer CSC to be retrieved from endosomes to the Golgi.^5^, ^6^ Loss of retromer CSC function through knockdown or genetic knockout results in mislocalization of the CIMPR to endosomes and its subsequent degradation in lysosomes. We also reported that mistrafficking of the CIMPR results in a failure to deliver lysosomal hydrolases such as Cathepsin D to the lysosome—a predictable outcome if the retrieval of the CIMPR is impaired.

## 3 IMPORTANT ADVANCES

Thus, early on in the studies of the retromer complex in mammals, it was established that the retromer CSC associates with the tail of the CIMPR to mediate its endosome‐to‐Golgi retrieval. The motif in the CIMPR tail that is required for retromer‐mediated endosome‐to‐Golgi retrieval was identified through a tried‐and‐tested approach employing CD8 reporters and mutagenesis of the CD8‐CIMPR construct. That study identified a sequence comprising Trp‐Leu‐Met (WLM) in the CIMPR tail as necessary for its retrieval.^9^ Interestingly, the reports from the Cullen and Steinberg labs reveal that the SNX‐BAR dimer associates with the CIMPR tail via the WLM motif.^3^, ^4^

Over time, the functioning of retromer‐mediated endosome‐to‐Golgi retrieval has become better understood. A major advance by the Cullen laboratory was the identification of SNX5 and SNX6 as retromer components and the demonstration that these two SNX‐BAR proteins could associate with dynein by binding to dynactin.^10^, ^11^ Subsequently, it was recognized that mammalian retromer, unlike the complex in yeast, is not a stable heteropentamer but a much looser association of the retromer CSC with the SNX‐BAR dimer.^12^ Thus, the retromer CSC requires the Rab7a and Snx3 proteins for its membrane association, whereas the SNX‐BAR dimer can associate with endosomes through the phox‐homology (PX) domains that bind to phosphotidyl inositol 3‐phosphate.^13^, ^14^, ^15^, ^16^ The mammalian retromer CSC, unlike the yeast CSC, associates with a collection of accessory proteins, for example, the WASP and Scar Homolog (WASH) complex that is recruited to endosomes through an interaction between the Vps35 protein and the Fam21 subunit of the WASH complex.^17^, ^18^, ^19^, ^20^ The patho‐physiological importance of the retromer‐WASH complex interaction is underscored by the fact that a Parkinson’s disease (PD)‐causing mutation in Vps35 impairs the binding of the WASH complex to the retromer CSC resulting in less endosomally localized WASH complex leading to trafficking defects.^21^, ^22^, ^23^

## 4 OUT WITH THE OLD, IN WITH THE NEW?

The recent reports from the Cullen and Steinberg labs have suggested that the role of the retromer CSC in sorting the CIMPR for retrieval to the Golgi is questionable because they could not show any mislocalization of the CIMPR when components of the retromer CSC are silenced by RNAi or deleted by targeted knockout. They cite the studies from my lab and that of Bonifacino^5^, ^6^ as the sources for the evidence that the retromer CSC is necessary for the retrieval of the CIMPR but of course there are other published studies that agree with those early papers. There are the data from Bulankina et al that was part of their investigation into the function of the TIP47 protein^24^ and the study of Hao et al confirmed the requirement for the retromer CSC to mediate the endosome‐to‐Golgi retrieval of the CIMPR in their analysis of WASH complex regulating machinery.^25^

Could the differences in effects on CIMPR trafficking boil down to different assays—what is the best way to measure the endosome‐to‐Golgi retrieval of the CIMPR? There is no definitive answer to that question and different labs have employed an array of techniques that are centred on the use of microscopy to visualize the CIMPR. The assay used by Hao et al^25^ measures the dispersal of the CIMPR to peripheral structures and has been used previously in retromer‐focussed studies.^26^ It was suggested however that the CIMPR dispersal assay employed by Hao et al may be insufficiently robust to properly determine the effect of loss of retromer CSC function on the localization of the CIMPR.^4^ Curiously, one of the reports to show that the retromer CSC is required for CIMPR retrieval was when the role of SNX5 and SNX6 in CIMPR retrieval was identified using a CIMPR dispersal assay.^10^ On that occasion knockdowns of Vps35, Vps29 or Vps26 all impaired CIMPR retrieval in a manner similar to a SNX5 or SNX6 knockdown. My own lab generally favours the use of automated microscopy as a means of measuring endosome‐to‐Golgi retrieval to avoid potential bias introduced at the time of imaging. Using such an assay, we have shown that loss of Snx3, Rab7a, Vps26a, Vps35 or SNX1 function all cause a significant endosome‐to‐Golgi retrieval defect of a CD8‐CIMPR reporter protein.^17^, ^27^ Another way to measure CIMPR retrieval to the TGN is the application of immunofluorescence with Pearson’s correlation—the technique employed in the paper by Kvainickas et al, where they report no role for the retromer CSC in CIMPR retrieval.^4^ In a surprising volte‐face, the study by Kvainickas et al stands in stark contrast with a previous report where it was shown that the PD‐causing Vps35 mutant was equivalent to a Vps35 null with respect to impairing CIMPR retrieval to the TGN using immunofluorescence and Pearson’s correlation to measure retrieval.^22^

## 5 CONCLUDING REMARKS

The finding that the SNX‐BAR dimer can associate with the tail of the CIMPR is undoubtedly a significant advance in the understanding of how the endosome‐to‐Golgi retrieval of the CIMPR occurs. But does the observation that the SNX‐BAR dimer can bind the CIMPR tail negate the role of the retromer CSC? There is sufficient evidence, accumulated by labs operating independently (including studies authored by Cullen and Steinberg^10^, ^22^), that the retromer CSC is required for the retrieval of the CIMPR to argue that it has a major role—possibly by directly associating with the CIMPR tail.^5^, ^6^, ^9^, ^10^, ^14^, ^17^, ^22^, ^24^, ^25^, ^28^ What then is the truth? Without further experimentation the answer to that question will have to wait. I suspect however that the “truth” will end up being a version of both models where the tail of the CIMPR can associate with the retromer CSC, possibly to ensure it is concentrated, albeit briefly, in endosomal microdomains that may require the presence of WASH‐complex generated F‐actin. Following the concentration of the CIMPR in the endosomal membrane, the SNX‐BAR proteins may bind and direct the CIMPR into a tubule for retrieval to the Golgi. Proteins such as RME‐8 that can associate with both the SNX‐BAR dimer and the WASH complex^22^, ^29^, ^30^, ^31^ may coordinate the transfer of cargo such as the CIMPR from the retromer CSC to the SNX‐BAR dimer. Both sets of proteins then will have a direct role in the CIMPR retrieval but the relative importance of the retromer CSC and SNX‐BAR dimer may vary from cell‐to‐cell and tissue‐to‐tissue according to the primary function of the cell(s) in question.

## References

[1] Burd C , Cullen PJ . Retromer: a master conductor of endosome sorting. Cold Spring Harb Perspect Biol. 2014;6(2):1–13. 10.1101/cshperspect.a016774 PMC3941235 24492709

[2] Seaman MN . The retromer complex—endosomal protein recycling and beyond. J Cell Sci. 2012;125:4693–4702. 23148298 10.1242/jcs.103440 PMC3517092

[3] Simonetti B , Danson CM , Heesom KJ , Cullen PJ . Sequence‐dependent cargo recognition by SNX‐BARs mediates retromer‐independent transport of CI‐MPR. J Cell Biol. 2017;216:3695–3712. 28935633 10.1083/jcb.201703015 PMC5674890

[4] Kvainickas A , Jimenez‐Orgaz A , Nägele H , Hu Z , Dengjel J , Steinberg F . Cargo‐selective SNX‐BAR proteins mediate retromer trimer independent retrograde transport. J Cell Biol. 2017;216:3677–3693. 28935632 10.1083/jcb.201702137 PMC5674888

[5] Arighi CN , Hartnell LM , Aguilar RC , Haft CR , Bonifacino JS . Role of the mammalian retromer in sorting of the cation‐independent mannose 6‐phosphate receptor. J Cell Biol. 2004;165:123–133. 15078903 10.1083/jcb.200312055 PMC2172094

[6] Seaman MN . Cargo‐selective endosomal sorting for retrieval to the Golgi requires retromer. J Cell Biol. 2004;165:111–122. 15078902 10.1083/jcb.200312034 PMC2172078

[7] Seaman MN , McCaffery JM , Emr SD . A membrane coat complex essential for endosome‐to‐Golgi retrograde transport in yeast. J Cell Biol. 1998;142:665–681. 9700157 10.1083/jcb.142.3.665 PMC2148169

[8] Nothwehr SF , Ha SA , Bruinsma P . Sorting of yeast membrane proteins into an endosome‐to‐Golgi pathway involves direct interaction of their cytosolic domains with Vps35p. J Cell Biol. 2000;151:297–310. 11038177 10.1083/jcb.151.2.297 PMC2192648

[9] Seaman MN . Identification of a novel conserved sorting motif required for retromer‐mediated endosome‐to‐TGN retrieval. J Cell Sci. 2007;120:2378–2389. 17606993 10.1242/jcs.009654

[10] Wassmer T , Attar N , Bujny MV , Oakley J , Traer CJ , Cullen PJ . A loss‐of‐function screen reveals SNX5 and SNX6 as potential components of the mammalian retromer. J Cell Sci. 2007;120:45–54. 17148574 10.1242/jcs.03302

[11] Wassmer T , Attar N , Harterink M , et al. The retromer coat complex coordinates endosomal sorting and dynein‐mediated transport, with carrier recognition by the trans‐Golgi network. Dev Cell. 2009;17:110–122. 19619496 10.1016/j.devcel.2009.04.016 PMC2714578

[12] Swarbrick JD , Shaw DJ , Chhabra S , et al. VPS29 is not an active metallo‐phosphatase but is a rigid scaffold required for retromer interaction with accessory proteins. PLoS One. 2011;6(5):e20420. 21629666 10.1371/journal.pone.0020420 PMC3101248

[13] Rojas R , van Vlijmen T , Mardones GA , et al. Regulation of retromer recruitment to endosomes by sequential action of Rab5 and Rab7. J Cell Biol. 2008;183:513–526. 18981234 10.1083/jcb.200804048 PMC2575791

[14] Seaman MN , Harbour ME , Tattersall D , Read E , Bright N . Membrane recruitment of the cargo‐selective retromer subcomplex is catalysed by the small GTPase Rab7 and inhibited by the Rab‐GAP TBC1D5. J Cell Sci. 2009;122:2371–2382. 19531583 10.1242/jcs.048686 PMC2704877

[15] Harterink M , Port F , Lorenowicz MJ , et al. A SNX3‐dependent retromer pathway mediates retrograde transport of the Wnt sorting receptor Wntless and is required for Wnt secretion. Nat Cell Biol. 2011;13:914–923. 21725319 10.1038/ncb2281 PMC4052212

[16] Vardarajan BN , Bruesegem SY , Harbour ME , et al. Identification of Alzheimer disease‐associated variants in genes that regulate retromer function. Neurobiol Aging. 2012;33:2231.e15–2231.e30. 10.1016/j.neurobiolaging.2012.04.020 PMC3391348 22673115

[17] Harbour ME , Breusegem SY , Antrobus R , Freeman C , Reid E , Seaman MN . The cargo‐selective retromer complex is a recruiting hub for protein complexes that regulate endosomal tubule dynamics. J Cell Sci. 2010;123:3703–3717. 20923837 10.1242/jcs.071472 PMC2964111

[18] Harbour ME , Breusegem SY , Seaman MN . Recruitment of the endosomal WASH complex is mediated by the extended 'tail' of Fam21 binding to the retromer protein Vps35. Biochem J. 2012;442:209–220. 22070227 10.1042/BJ20111761

[19] Jia D , Gomez TS , Billadeau DD , Rosen MK . Multiple repeat elements within the FAM21 tail link the WASH actin regulatory complex to the retromer. Mol Biol Cell. 2012;23:2352–2361. 22513087 10.1091/mbc.E11-12-1059 PMC3374753

[20] Helfer E , Harbour ME , Henriot V , et al. Endosomal recruitment of the WASH complex: active sequences and mutations impairing interaction with the retromer. Biol Cell. 2013;105:191–207. 23331060 10.1111/boc.201200038

[21] Zavodszky E , Seaman MN , Moreau K , et al. Mutation in VPS35 associated with Parkinson's disease impairs WASH complex association and inhibits autophagy. Nat Commun. 2014;5:3828. 24819384 10.1038/ncomms4828 PMC4024763

[22] McGough IJ , Steinberg F , Jia D , et al. Retromer binding to FAM21 and the WASH complex is perturbed by the Parkinson disease‐linked VPS35(D620N) mutation. Curr Biol. 2014;24:1678. 28903028 10.1016/j.cub.2014.07.004 PMC5628949

[23] Follett J , Norwood SJ , Hamilton NA , et al. The Vps35 D620N mutation linked to Parkinson's disease disrupts the cargo sorting function of retromer. Traffic. 2014;15:230–244. 24152121 10.1111/tra.12136

[24] Bulankina AV , Deggerich A , Wenzel D , et al. TIP47 functions in the biogenesis of lipid droplets. J Cell Biol. 2009;185:641–655. 19451273 10.1083/jcb.200812042 PMC2711566

[25] Hao YH , Doyle JM , Ramanathan S , et al. Regulation of WASH‐dependent actin polymerization and protein trafficking by ubiquitination. Cell. 2013;152:1051–1064. 23452853 10.1016/j.cell.2013.01.051 PMC3640276

[26] Gomez TS , Billadeau DD . A FAM21‐containing WASH complex regulates retromer‐dependent sorting. Dev Cell. 2009;17:699–711. 19922874 10.1016/j.devcel.2009.09.009 PMC2803077

[27] Breusegem SY , Seaman MN . (2014). Genome‐wide RNAi screen reveals a role for multipass membrane proteins in endosome‐to‐golgi retrieval. Cell Rep. 2014;9:1931–1945. 25464851 10.1016/j.celrep.2014.10.053 PMC4542293

[28] Fjorback AW , Seaman M , Gustafsen C , et al. Retromer binds the FANSHY sorting motif in SorLA to regulate amyloid precursor protein sorting and processing. J Neurosci. 2012;32:1467–1480. 22279231 10.1523/JNEUROSCI.2272-11.2012 PMC6796259

[29] Shi A , Sun L , Banerjee R , Tobin M , Zhang Y , Grant BD . Regulation of endosomal clathrin and retromer‐mediated endosome to Golgi retrograde transport by the J‐domain protein RME‐8. EMBO J. 2009;28:3290–3302. 19763082 10.1038/emboj.2009.272 PMC2776105

[30] Popoff V , Mardones GA , Bai SK , et al. Analysis of articulation between clathrin and retromer in retrograde sorting on early endosomes. Traffic. 2009;10:1868–1880. 19874558 10.1111/j.1600-0854.2009.00993.x

[31] Freeman CL , Hesketh G , Seaman MN . RME‐8 coordinates the activity of the WASH complex with the function of the retromer SNX dimer to control endosomal tubulation. J Cell Sci. 2014;127:2053–2070. 24643499 10.1242/jcs.144659 PMC4004978

